# Factors Associated with Bovine Neonatal Pancytopenia (BNP) in Calves: A Case-Control Study

**DOI:** 10.1371/journal.pone.0034183

**Published:** 2012-05-11

**Authors:** Sarah L. Lambton, Adrian D. Colloff, Richard P. Smith, George L. Caldow, Sandra F. E. Scholes, Kim Willoughby, Fiona Howie, Johanne Ellis-Iversen, Graham David, Alasdair J. C. Cook, Andrew Holliman

**Affiliations:** 1 Animal Health and Veterinary Laboratories Agency, Addlestone, Surrey, England; 2 Animal Health and Veterinary Laboratories Agency, Truro, Cornwall, England; 3 Veterinary Services, Scottish Agricultural College Consulting, St Boswells, Scottish Boarders, Scotland; 4 Animal Health and Veterinary Laboratories Agency, Lasswade, Penicuik, Midlothian, Scotland; 5 Moredun Research Institute, Penicuik, Midlothian, Scotland; 6 Veterinary Services, Scottish Agricultural College Consulting, Penicuik, Midlothian, Scotland; 7 Department for Environment, Food and Rural Affairs (Defra), London, England; 8 Animal Health and Veterinary Laboratories Agency, Penrith, Cumbria, England; Institute for Animal Health, United Kingdom

## Abstract

Bovine neonatal pancytopenia (BNP; previously known as idiopathic haemorrhagic diathesis and commonly known as bleeding calf syndrome) is a novel haemorrhagic disease of young calves which has emerged in a number of European countries during recent years. Data were retrospectively collected during June to November 2010 for 56 case calves diagnosed with BNP between 17 March and 7 June of the same year. These were compared with 58 control calves randomly recruited from herds with no history of BNP. Multivariable logistic regression analysis showed that increased odds of a calf being a BNP case were associated with its dam having received PregSure® BVD (Pfizer Animal Health) vaccination prior to the birth of the calf (odds ratio (OR) 40.78, p<0.001) and its herd of origin being located in Scotland (OR 9.71, p = 0.006). Decreased odds of a calf being a BNP case were associated with the calf having been kept outside (OR 0.11, p = 0.006). The longer that a cattle herd had been established on the farm was also associated with decreased odds of a calf in that herd being a BNP case (OR 0.97, p = 0.011).

## Introduction

Bovine neonatal pancytopenia (BNP) was previously known as idiopathic haemorrhagic diathesis and is commonly known as bleeding calf syndrome. It is a novel haemorrhagic disease of young calves which has emerged in a number of European countries during recent years. This disease has been reported across Great Britain (GB) [1], [2], in the Republic of Ireland [3] and in a number of countries on mainland Europe [4].

BNP typically affects calves younger than one month old, independent of breed or sex. Clinical signs in affected calves may include cutaneous haemorrhage, petechiation of mucosae, melaena and pyrexia, although some affected calves are found dead without clinical signs being observed. The typical haematological finding is thrombocytopenia, which may be accompanied by neutropenia, lymphopenia and non-regenerative anaemia. Histopathology of the bone marrow of affected calves reveals trilineage hypoplasia involving simultaneous, extensive, pronounced reduction in all three main haematopoietic cell series. The haematological and pathological findings suggest that destruction of bone marrow progenitor cells leads to hypoplastic pancytopenia and consequently haemorrhage due to thrombocytopenia [5]. On affected farms, the incidence of clinical cases is generally low but the case fatality rate is very high and can become important at the individual farm level, with losses of up to 5% of calves in a herd being reported [4], [5].

Investigations into the aetiology of BNP are on-going in a number of countries. A German study reported the detection of circovirus DNA in 5 out of 25 cases of BNP but also in 1 out of 8 unaffected control calves [6]; other studies have found no evidence to suggest a viral aetiology for BNP [4], [7]. Limited studies have been unable to demonstrate a simple mode of inheritance by investigation of a mutation in factor XI [8] or by investigation of major histocompatibility complex allelic frequencies [9]. It has been observed that BNP can be induced in some calves by ingestion of colostrum harvested from cows which have previously given birth to calves which developed BNP [10], and it has been suggested that alloantibodies from colostrum may play a crucial role in the pathogenesis of BNP [11]. Sera of PregSure® BVD (Pfizer Animal Health) vaccinated BNP dams have been shown to contain alloreactive leukocyte-binding antibodies and these alloantibodies cross-react with the bovine kidney cell line used for PregSure® BVD production [12]. There is also evidence that some PregSure® BVD induced alloantibodies recognise cattle major histocompatibility complex class I antigen [13]. Nonetheless, the exact aetiology and pathophysiology of BNP remains to be fully elucidated.

In order to investigate common factors in calves developing BNP, a case-series study of 75 clinical cases of BNP was conducted by the Veterinary Laboratories Agency (VLA) and Scottish Agricultural College (SAC) during 2009 [14]. This study also provided material to investigate the potential involvement of infectious agents or other causes of bone marrow suppression and, through a defined necropsy protocol, to provide a definitive histopathological description of the disease [15]. Factors which occurred in more than 60% of case calves in this study included vaccination of the calf’s dam with at least one vaccine during the 12 month period prior to the birth of the calf of interest or the 4 week period thereafter, ingestion of maternal colostrum, addition of cattle to the herd during the previous 12 months and the presence of sheep on the farm. Case-series studies can provide a useful insight into factors that may be associated with a novel disease, but only provide weak evidence due to the absence of a set of control animals for comparison. These preliminary findings were therefore used to inform the design of the case-control study reported here, which aimed to investigate the association between putative risk factors and clinical BNP in calves in GB.

## Methods

A case-control study was designed to estimate the strength of association between putative risk factors and the occurrence of clinical BNP in calves in GB. Potential risk factors were selected based on information from a previous case-series study [14] and from the available literature and expert opinion at that time. The study was carried out by the VLA and SAC, both delivery agents for Government farm animal disease scanning surveillance in GB. As part of this function, the VLA and SAC provide a laboratory-based farm animal diagnostic service, including necropsies, to private veterinary surgeons (PVSs).

### Sample Criteria

The sampling frame for this study was the British calf population; the unit of study was defined as the individual calf. However, since potential risk factors were both animal-level and herd-level, recruitment for this study was restricted to a single calf per farm. In order to facilitate accurate data collection, calves were ineligible for the study if they had moved from one herd to another, and thus were no longer in their birth herd.

### Case Definition

A case was defined as a calf 28-days-old or younger, with unexplained haemorrhages detected at necropsy, and trilineage hypoplasia of bone marrow confirmed by histopathology. Trilineage hypoplasia was defined as concurrent depletion of erythroid and myeloid cells and megakaryocytes from bone marrow resulting in less than 25% cellularity [15]. A notional date of enrolment into the study, for each case, was defined as the date on which the necropsy was carried out.

#### Control definition

For each case calf, a control calf, matched by purpose of herd of origin i.e. beef cow herd or dairy herd, was selected at random from the British calf population. Thus the cases and controls were frequency matched to include the same proportions of calves from beef cow and dairy herds. A control was defined as a calf that had never shown any evidence of clinical disease.

It was a requirement that control calves were aged between 28 and 56-days-old on the notional date of enrolment of the case calf. This age range ensured that control calves had passed through the ‘at risk’ period for development of BNP (7 to 28-days-old), and allowed a margin for the possibility of late onset cases. Linking control calf age with the notional date of enrolment for the case calf ensured a similar representation of cases and controls born in each month throughout the study period; thus control calves represented the ‘at risk’ population during the same period that the case calves were enrolled. Furthermore, it minimised potential differences in recall bias between cases and controls.

Control calves were recruited from herds with at least 100 adult (≥2 years old) female cattle present. It was a requirement that, at the time of enrolment and through to the end of the study period, herds providing control calves had no history of a diagnosis of BNP and no history of calves:

- <29-days-old presenting with apparent spontaneous haemorrhage from the skin or with excessive or prolonged haemorrhage from wound sites such as ear tag wounds and injection sites (from 1 January 2007);
- <29-days-old presenting with melaena (from 1 January 2009);
- 7 to 30-days-old found dead in the absence of premonitory clinical signs (from 1 September 2009).

#### Exclusion criteria

Exclusion criteria were implemented to further ensure that there were no unmonitored animals in a ‘control herd’, that could have shown signs of disease as described above, and which would therefore have led to exclusion of a selected calf as a control. Calves were considered ineligible if they originated from herds in which, from 1 January 2009 through to the end of the study period, any healthy male calves in the herd were humanely slaughtered before they reached 29-days-old or any calves (male or female) were sold before they reached 29-days-old. To ensure that case and control calves were recruited from the same general population of calves, these criteria were also applied to case calves.

### Enrolment

#### Case selection

Case enrolment took place retrospectively from June to November 2010. Case calves were identified through voluntary submission of animals to VLA Regional Laboratories and SAC Disease Surveillance Centres for necropsy. Such submissions were encouraged by the offer of free necropsy examination of suspect cases. The owners of all herds with case calves diagnosed between 17 March and 7 June 2010 were randomly ordered and contacted, initially by telephone, to seek their consent to participate in the study. If multiple case calves had been detected in a selected herd at the time of contact, then a single case calf was selected at random. Once a calf had been recruited from a herd, no further calves from that herd were eligible for inclusion in the study.

#### Control selection

Control calves were selected from beef cow and dairy herd population data, obtained using the Rapid Analysis and Detection of Animal-related Risks (RADAR) information management system [16]. RADAR accesses information from the Cattle Tracing System, which records the identification, death and movements of all cattle issued with passports from 1998 onwards, and all movements of older cattle. From this, a randomly ordered list of herds (excluding known ‘case herds’) was produced. Herds on this list were contacted in sequential order by a VLA staff member and assessed for willingness to participate and eligibility. If a herd was not willing to participate or was ineligible, the next herd on the list (of the same herd purpose) was contacted. Once a potential ‘control herd’ was identified, the youngest calf in that herd, meeting the control definition, was identified as the control calf.

### Sample Size

A minimum sample size of 56 case and 56 control calves was calculated using Win Episcope 2.0 [17] to provide 95% confidence of detecting an odds ratio (OR) of 3 or more provided the exposure rate amongst controls lay between 28 and 47% (designed to encompass exposures to a range of potential risk factors in the national herd). This calculation was predicated on univariable analyses; risk factors which were found significant at the univariable stage would be further investigated through multivariable analyses.

### Data Collection

Data collection took place during June to November 2010. VLA and SAC Veterinary Investigation Officers (VIOs) conducted personal interviews with the owners of case and control calves, or with a suitable representative of the owner, using a standard questionnaire (Information S1). The questionnaire captured information on a number of herd-level and animal-level exposures, and is summarised in Table 1.

**Table 1:** Information collected in the study questionnaire.

Level	Information
Herd details	Location and cattle farming history.
	Organic/standard/converting.
	Size and composition.
	Cattle movements onto the farm, since 2007.
	Contact with cattle on other farms, since 2007.
	Contact with sheep on this and other farms, since 2007.
Calf details	Date of birth and sex.
	Colostrum management.
	Housing.
Dam details	Date of birth and time on the farm.
	Contact with cattle on other farms, while pregnant with the calf of interest.
	Contact with sheep on this and other farms, whilst pregnant with the calf of interest.
	Diet.
	Vaccination history (including dates and batch numbers for all vaccines used) since the start of 2005.
Other	Dam and sire breed.

In order to validate the reported dam vaccination histories, the VIOs conducting the interviews scrutinised each farm’s Animal Medicine Record Book, if this record was available at the time of the interview. All farmers in GB are required by law to record the administration of all medicines to food producing animals. Each owner was also asked to authorise disclosure by their PVS of vaccine sales records for the herd. For each herd with an incomplete on-farm written or electronic record of the dam vaccination history in question, the relevant vaccine sales records were obtained from the herd’s PVS. There is evidence that BNP results from a factor present in the colostrum of certain cows [10], so reports of vaccination were only included in the dataset if the vaccination had been administered to the dam before the birth of the calf of interest.

Subsequent to the data collection interview, the owners of control calves were telephoned fortnightly by VLA and SAC VIOs. If any control became ineligible before the end of the study period, that calf and herd were rejected and replaced with a new eligible calf, from a new herd.

### Analysis

All statistical analyses were carried out in Stata 10.0 (Statacorp, College Station, TX). All variables were entered individually into a univariable logistic regression model. However, since the cases and controls were frequency matched on herd purpose, in each step the variable ‘herd purpose’ (beef cow herd or dairy herd) was included in the model, so that ORs calculated were adjusted for the stratification in the data. Variables were selected for inclusion in a multivariable model if p<0.1, in order to ensure that variables of interest were not omitted.

Subsequently, all variables significant in univariable analyses were entered into a multivariable logistic regression model and a backwards stepwise logistic regression was carried out; the variable with the highest p-value was removed and the model re-run. This process was repeated until all variables remaining in the model were significant (using a more stringent critical value of p<0.05). The possibility of collinearity between predictor variables was addressed initially by seeking correlations between the explanatory variables and, following the model fitting, by stepwise removal of variables to assess the effect on the remaining variable coefficients. Furthermore, after model fitting, variance inflation factors (VIF) [18] were calculated and examined. Model fit was examined using Hosmer & Lemeshow’s goodness of fit test and a linktest [18]. These methods, in combination with likelihood ratio tests as appropriate, were used to compare logistic regression models to clustered, random effects (using herd purpose as a cluster/random effect variable) and conditional logistic regression models (treating cases and controls as individually matched) to ensure that the final model was the most suitable for the data structure.

Where appropriate, and if further information was available, variables significant in the final model were subject to further analyses, in order to better understand the nature of the association between BNP and each risk factor.

## Results

### Descriptive Statistics

During the study period, 56 eligible case calves were identified and enrolled into the study. A total of 312 potential ‘control herds’ were contacted; of these herds, 33% did not respond to any contact, 31% declined and 18% were found to be ineligible. Ineligible herds were mainly those selling calves less than 29-days-old or humanely slaughtering healthy male calves less than 29-days-old (17 farms) or herds that had calves with a clinical presentation requiring exclusion (13 farms). This provided a total of 58 eligible control calves. A Yates-corrected chi-squared test was used to evaluate any differences in participation between ‘control herds’ in Scotland and ‘control herds’ in England and Wales. A higher percentage of Scottish dairy herds (67%) declined to participate in the study compared with English or Welsh dairy herds (25%; p<0.05). A higher percentage of Scottish beef cow herds (20%) were ineligible for the study compared with English or Welsh beef cow herds (7%; p<0.05).

Data were collected for the 56 eligible case calves and the 58 eligible control calves. One control calf was rejected when BNP was diagnosed in its herd during the study period, and a replacement calf was recruited from another herd. During March, April, May and June, 19, 55, 38 and 2 calves were enrolled into the study, respectively. One control calf was 57-days-old on its notional date of enrolment, but was retained in the study due to time constraints on data collection.

Table 2 shows, by case/control status and herd purpose, the number of herds recruited in England and Wales, and Scotland, and the mean total herd sizes and number of calves. Mean dam age was 6.2 years and 5.5 years for control and case calves respectively, and there was no evidence of any significant difference between them.

**Table 2:** Breakdown of the characteristics of the herds included in the study.

		Number of herds		Mean number of animals per herd	
Group	Herd type	Scotland	England & Wales	Calves	Total cattle
Case	Beef cow	44	6	132.4	450.1
	Dairy	0	6	94.8	398.8
Control	Beef cow	24	25	133.0	407.9
	Dairy	0	9	91.3	360.1

Of the 114 calves in the study, 111 received colostrum from their dam. Of the three that did not receive colostrum from their dam, one received pooled colostrum (case), one received pooled colostrum and ‘other’ colostrum (case), and one received ‘other’ colostrum (control). One calf that received colostrum from its dam also received commercial colostrum (control).

### Final Model

The variables remaining in the model after stepwise logistic regression are shown in Table 3. There were four missing values in the ‘PregSure® BVD use’ variable and one missing value in the ‘years cattle herd established on farm’ variable. Therefore the model is based on 109 data points, of which 54 were control and 55 were case calves. All VIF values were <2 (mean: 1.35), thus collinearity was not considered a problem [18]. Using Hosmer and Lemeshow’s goodness of fit test [18], the model fit was satisfactory (p = 0.247) and the linktest for specification error was not significant (p = 0.674). No other type of model improved the fit of the data.

**Table 3:** Final logistic regression model of the factors significantly associated with a change in the odds of a calf being a BNP case.

Variable	OR	p wald	95% CI
Years cattle herd established on the farm	0.97	0.011	0.94, 0.99
Herd in Scotland (Yes, 67:No, 42)	9.71	0.006	1.92, 49.14
Calf kept outside (Yes, 70:No, 39)	0.11	0.006	0.02, 0.53
PregSure® BVD administered to dam(Yes, 64:No, 45)	40.78	<0.001	10.14, 163.91

The odds of a calf being a BNP case decreased if the calf had been kept outside. Housing types were not mutually exclusive, as animals could have been kept both inside and out, therefore the variable ‘calf kept outside’ was explored in more detail using a variable with three categories: calf kept outside only (n = 15); calf housed indoors only (n = 39); calf kept both indoors and outside at some stage (n = 55). In this instance, the variable remained significant (Χ^2^ = 10.12, df = 2, p = 0.006) with the odds of disease being reduced in calves kept outside only and also calves kept indoors and outside, compared with those housed indoors only (ORs of 0.06 and 0.13 respectively).

The odds of a calf being a BNP case decreased as the number of years for which the cattle herd had been established on the farm increased. Herds with case calves had been established for a mean of 30.9 years (CI 24.9, 36.8) and herds with control calves had been established for a mean of 40.4 years (CI 33.6, 47.2).

The odds of a calf being a BNP case increased if the herd was in Scotland and also increased if PregSure® BVD had been administered to the calf’s dam at any time from the start of 2005 through to the birth of the calf. The dams of 50 of the 55 cases had received PregSure® BVD, compared with 14 of the 54 dams of controls. In order to better understand the association with BNP, the ‘PregSure® BVD use’ variable was examined in more detail. A new variable with four categories was created: no Bovine Viral Diarrhoea Virus (BVDV) vaccine; vaccinated with PregSure® BVD; vaccinated with PregSure® BVD plus other BVDV vaccine; vaccinated with other BVDV vaccine. This variable was significant when it replaced overall PregSure® BVD use in the model presented in Table 3 (Χ^2^ = 43.60, df = 3, p<0.001); both the second and third categories, which included PregSure® BVD use, were associated with an increase in the odds of a calf being a BNP case (ORs of 39.9 and 33.4 respectively). The original binary ‘PregSure® BVD use’ variable was retained in the model as it was more parsimonious.

PregSure® BVD may have been used at any time from 2005, when the vaccine was first marketed in the UK. In 2005, 2006, 2007, 2008, 2009 and 2010, a total of 1, 13, 30, 52, 53 and 32 dams respectively were vaccinated with PregSure® BVD. When inserted separately into the final model, replacing overall PregSure® BVD use, an increase in the odds of a calf being a BNP case was significantly associated with PregSure® BVD use in each of 2007, 2008, 2009 and 2010 (p<0.001) and showed a trend in the same direction for 2006 (p = 0.076). Although there is likely to have been some collinearity between these variables, there was no evidence to suggest that PregSure® BVD use in any particular year was more significant.

Time since last vaccination of the dam with PregSure® BVD, relative to the birth date of the calf of interest (included as a continuous explanatory variable), was not significantly associated with the likelihood of a calf developing BNP, either replacing overall PregSure® BVD use in the final model, or in a bivariable model stratified by herd purpose. An explanatory variable comprising four categories was created: never used PregSure® BVD; PregSure® BVD used before/on 31 December 2008; PregSure® BVD used on/after 1January 2009; PregSure® BVD used both before/on 31 December 2008 and on/after 1January 2009. PregSure® BVD use was associated with significantly higher odds of a calf being a BNP case in all categories (Χ^2^ = 62.78, df = 3, p<0.001), compared to never having used PregSure® BVD; however, none of the ‘PregSure® BVD use’ categories differed significantly from each other (ORs of 14.0, 7.8 and 62.8; CI (1.3, 149.2), (1.4, 41.9) and (16.9, 234.3) respectively).

The number of doses of PregSure® BVD (total number of times PregSure® BVD was administered to the dam) was examined; the assumption was made that on each occasion a full dose was administered. In a bivariable model using number of doses as a categorical variable, stratified by herd purpose, all numbers of doses of PregSure® BVD were significantly associated with an increase in the odds of a calf being a BNP case (Χ^2^ = 57.20, df = 4, p<0.001), but no number of doses differed significantly from any other (Table 4). Dose was not considered to be a robust predictor variable since we did not have a full lifetime vaccination history for every dam.

**Table 4:** Breakdown of numbers of PregSure® BVD doses and their association with the likelihood of a calf being a BNP case.

	Number of:			
No. of PregSure® BVD doses	Cases	Controls	OR	95% CI
0	5	40	1.0	n/a
1	4	1	45.3	3.7, 561.3
2	7	5	12.0	2.7, 53.8
3	16	3	47.1	9.7, 228.7
4+	24	5	38.1	9.9, 146.5

The data were examined for any evidence of bias amongst cases or controls in the reporting of PregSure® BVD use. The number of years for which we had information on PregSure® BVD use for each dam was examined as a proportion of all years for which PregSure® BVD may have been used in each dam. This did not differ significantly between the dams of cases and controls (mean proportion: 0.97 and 0.91 respectively, p = 0.233).

Where dam vaccine records were not corroborated by an on-farm written or electronic record, farmers’ PVSs were contacted to obtain a copy of the relevant vaccine sales record. In only one case did this affect the ‘PregSure® BVD use’ variable. PVS records suggested that the owner of one case herd may have been sold an alternative BVDV vaccine rather than PregSure® BVD, while the initial record, based on owner recall, indicated PregSure® BVD had been used. Sales records cannot confirm that the dam in question was vaccinated with the alternative vaccine; however, when the ‘PregSure® BVD use’ variable was adjusted to reflect this change and tested in the final model, it did not affect any other variables in the model, and PregSure® BVD use remained significant (OR 33.71; CI 8.72, 130.24; p<0.001).

## Discussion

In this study, data were collected for 56 case calves and 58 control calves. The study design required that only one calf was recruited from any particular herd. Case and control notional dates of enrolment were evenly distributed throughout the spring in 2010 (from 17 March to 7 June). There was no statistically significant difference in herd size between cases and controls, as the slightly larger size of case herds observed could easily have arisen by chance.

There is experimental evidence that a factor present in the colostrum of certain cows is implicated in the pathogenesis of BNP [10]. Therefore, it was important to ascertain the source of colostrum for calves in this study. Almost all (96%) of the calves in the study received colostrum only from their dam; consequently colostrum-derived factors will be accurately represented by the individual dam records collected.

As the sample size calculation was predicated on univariate analysis then our power to detect risk factors that might have been associated with an odds ratio below 3.0 is limited, particularly in multivariate analysis. Nonetheless, a number of important risk factors were observed. There was a strong association between BNP status and a history of dam vaccination with the PregSure® BVD vaccine, an inactivated BVDV type 1 vaccine. The odds of a calf being a BNP case were at least 10 times higher (lower 95% CI boundary) if its dam had been vaccinated with PregSure® BVD at any time prior to the calf’s birth. In every year except 2005, PregSure® BVD administration showed evidence of an association with BNP status, and there was no evidence that the strength of this association was notably different in any particular year, although there is some loss of power due to the reduced sample size in some years. There was no discernible effect of number of doses. There was no association between time since last vaccination with PregSure® BVD and BNP status; however, again there is a loss of power since relatively few of the dams of control calves (14 animals) had received PregSure® BVD. When time since last vaccination was examined as a categorical variable, no significant difference was observed between dams vaccinated in or after 2009 and dams vaccinated before 2009. An effect of number of occasions on which PregSure® BVD was administered may have been difficult to identify since a full lifetime PregSure® BVD vaccination history was not available for every dam; however, there was no apparent difference in the completeness of these records between cases and controls.

A potential link between PregSure® BVD and BNP has been suggested [10], [12], [13]; the vaccine was withdrawn from the European market in 2010 [19]. Our study provides strong epidemiological evidence of an association between PregSure® BVD and BNP, but a case-control study cannot definitively prove causation. An association between maternal vaccination and induction of disease via colostral antibody has been described previously in cattle, e.g. immune-mediated haemolytic anaemia in calves following the use of vaccines against anaplasmosis [20] and babesiosis [21], [22]. In the case of BNP, some cows produce alloantibodies which bind to calf peripheral leukocytes [11], [23] and such alloantibodies have been identified in the sera of PregSure® BVD vaccinated BNP dams [12]. It is plausible that these, or other induced alloantibodies, recognise a target present on neonatal haematopoietic stem cells, triggering their destruction and the development of trilineage hypoplasia in the bone marrow of calves, following ingestion of colostrum. This hypothesis could explain the development of the disease only in calves and not in the dam. Furthermore, alloantibodies directed against foetal lymphocyte antigens have been previously detected in sera of primiparous cattle [24] and the possibility that such antibodies may be elevated by dysregulation of the immunology of pregnancy following vaccination cannot be discounted. Further work to define the target antigen on neonatal haemopoietic stem cells is required and other factors to consider in future studies are the antigen, cell line and adjuvant employed in the vaccine preparation and the timing and frequency of vaccination.

The overall incidence of clinical BNP appears to be low in the context of the number of doses of PregSure® BVD sold across a number of European countries during recent years. The overall incidence for BNP at European Union level between 2004 and 2009 was estimated to be 0.016% based on a single dose of PregSure® BVD [25]. Consequently, it is possible that other factors play an important role in the aetiology of BNP and further studies to investigate these are required. However, although clinical BNP appears to be a low incidence disease, the occurrence of unrecognised subclinical disease also merits consideration. Better characterisation of the markers of disease in the preclinical stages should allow this effect to be investigated in experimental challenge systems and on case farms.

We have considered whether the association with PregSure® BVD identified by our study could be spurious due to confounding with the presence of active BVDV infection in the herds of interest; actively infected herds being possibly more likely to use BVDV vaccines. Although the BVDV infection status of the herds recruited into this study was not explicitly confirmed, a vaccine association was only identified with PregSure® BVD, despite three different BVDV vaccines having been used in GB during recent years. Furthermore, there is supportive temporal evidence for an association with PregSure® BVD; BNP was not identified in GB prior to the introduction of PregSure® BVD, although the other two BVDV vaccines were available. No evidence has been reported that the pathogenesis of BNP is associated with BVDV or other pestivirus (including Border disease virus) infection of affected calves despite having been extensively and explicitly sought [4], [7]. Virological investigations carried out by the VLA, SAC and Moredun Research Institute, involving screening 266 calves with histopathologically-confirmed trilineage hypoplasia of bone marrow, by real-time polymerase chain reaction, during 2009 and 2010, detected BVDV in only one of these calves (unpublished data). As BVDV infection is widespread in British cattle herds, it is to be expected that the virus would be found on occasions in tissues from calves that have died from causes not associated with BVDV infection. Finally, although BVDV-induced thrombocytopenia and associated haemorrhage have been reported in young calves [26], the pathogenesis of BVDV-induced thrombocytopenia is different to the pathogenesis of BNP. The characteristic bone marrow pathology underlying BNP is trilineage hypoplasia, consistent with an injury to pluripotential haematopoietic stem cells. The depletion of megakaryocytes leads to marked reduction in circulating thrombocytes and hence the haemorrhagic diathesis observed in calves with BNP. The lesion in calves affected with BNP is therefore distinctly different from the selective targeting of megakaryocytes and platelets that is a feature of pestivirus-induced thrombocytopenia in both BVD and classical swine fever [27].

There was an association with herd location; Scottish herds were 10 times more likely to contain a BNP calf than herds in England and Wales. Total calf births in Scotland in the spring of 2009 amounted to 276,352, compared to 571,219 in England and 124,430 in Wales. Although Scotland had more calves born per herd during the spring period (23 in Scotland, 12 in England and 10 in Wales), resulting in more calves per herd in the at-risk age group for BNP during this period, in absolute terms there were still more calves born in England and Wales during that time. Possible explanations for the association with herd nationality include greater awareness of BNP and its various clinical presentations amongst Scottish cattle farmers and greater motivation of Scottish cattle farmers to submit possible cases for investigation. It is also notable that compared with English and Welsh farmers, a higher percentage of the Scottish farmers who were contacted to request provision of a control calf, declined to participate. This could have biased control recruitment towards in English and Welsh herds. It is also possible that another, unknown, causal factor is more prevalent in Scotland.

The length of time for which the cattle herd had been established on the farm was also associated with a change in the odds of a calf being a BNP case. The likelihood of disease decreased by 3% with every additional year that the cattle herd had been present on the farm. Whilst it is possible to speculate on a range of plausible explanations, analysis of the study data did not offer any further insights and it is possible, especially in a small study that such an association has arisen purely by chance.

Calves kept outside at any point during their life were less likely to be BNP cases; calves that had been kept outside had 0.11 times the likelihood of being a case, compared with calves housed indoors only. It was thought that this could have been an artefact, since control calves had more opportunity to experience a variety of ‘housing’ types. However, calves that had been indoors and outside, and those that had been outside only, had a reduced chance of being a case compared with those housed indoors only, suggesting that a reduced risk of disease was associated with being kept outside. Calves outside may be less well monitored than calves indoors, resulting in a greater likelihood of BNP cases being missed due to lack of visibility or misidentification.

### Conclusions

This case-control study has shown a strong association between BNP and administration of PregSure® BVD to the dams of affected calves. This association is not proof of causation and further studies are required to elucidate the pathogenesis of the development of the haematopoietic abnormalities underlying BNP. Our power to detect any risk factors that might be more weakly associated with BNP is limited by the sample size and it is possible that presently unidentified factors are involved in the development of BNP in individual animals.

## Supporting Information

Information S1 Bovine neonatal pancytopenia case-control study questionnaire, used to collect the data presented in this paper.
(DOC)
