# Supplementary material for: Factors Associated with Bovine Neonatal Pancytopenia (BNP) in Calves: A Case-Control Study
Source: PLoS One. 2012 May 11;7(5):e34183. doi: 10.1371/journal.pone.0034183 (PMC3350487; doi:10.1371/journal.pone.0034183)
Supplement: Information S1 — Bovine neonatal pancytopenia case-control study questionnaire, used to collect the data presented in this paper. (DOC) [file pone.0034183.s001.doc]

**Supporting Information S1**


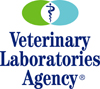


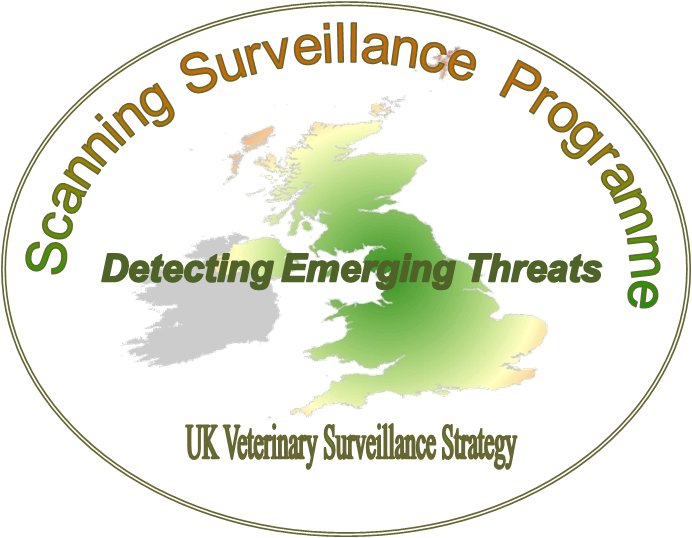


**BOVINE NEONATAL PANCYTOPENIA**

**Case-control Study Questionnaire**

**Produced by the Veterinary Laboratories Agency**

**For information please contact:**

**Mr A Holliman**

**VLA Penrith, Merrythought, Calthwaite, Penrith, Cumbria**

**CA11 9RR**

**Email: a.holliman@vla.defra.gsi.gov.uk**

## BOVINE NEONATAL PANCYTOPENIA INVESTIGATION

## CASE – CONTROL STUDY QUESTIONNAIRE

## PLEASE COMPLETE USING A BLACK PEN

It is imperative that complete and accurate data are obtained. Please ensure that answers are provided for every question. Where numerical answers are required do not leave any empty cells.

Please make a photocopy of the completed questionnaire and forward the photocopy to:

## Richard Smith

CERA (Area F)

## VLA Weybridge

## New Haw

## Addlestone

## Surrey

## KT15 3NB

Please retain the original completed questionnaire on file at your office.

| Date of interview: | D | D | M | M | Y | Y | Y | Y |
| --- | --- | --- | --- | --- | --- | --- | --- | --- |

| Time interview started: | HH : MM |  | Time interview ended: | HH : MM |
| --- | --- | --- | --- | --- |

| Name of person interviewed (BLOCK LETTERS): |  |
| --- | --- |

| VIO carrying out interview (BLOCK LETTERS): |  |
| --- | --- |

| Is this a case or control calf? **(circle correct response)** | CASE | CONTROL |
| --- | --- | --- |

**Question 1 – Farm/herd details**

| 1.1 | Name of owner:  (If different to interviewee) |  |
| --- | --- | --- |

| 1.2 | Address of main farm  premises: | Postcode: |
| --- | --- | --- |

| 1.3 | CPHH number: |  |  |  |  |  |  |  |  |  |  |  |
| --- | --- | --- | --- | --- | --- | --- | --- | --- | --- | --- | --- | --- |

| 1.4 | OS map reference of main farm premises: |  |  |  |  |  |  |  |  |
| --- | --- | --- | --- | --- | --- | --- | --- | --- | --- |

(Enter ‘0’ in first cell if not known)

| 1.5 | How long has the current cattle herd been established on this farm? (round up to the nearest whole year) | years |
| --- | --- | --- |

| 1.6 | Is the cattle herd an accredited organic herd? | Yes | No | Converting |
| --- | --- | --- | --- | --- |
|  | **(circle correct response)** |

**If yes**;

|  | How long has the cattle herd been accredited organic?  (round up to the nearest whole year) | years |
| --- | --- | --- |

**Question 2 – Herd composition**

| 2.1 | For the last 12 months what has been the average number of cattle kept on the farm for each type listed below? Enter approximate numbers; **do not** enter ranges of numbers. If a particular type of cattle is not kept on the farm enter ‘0’ as the number kept. |
| --- | --- |

| **Cattle Type** | **Description** | **Typical number kept in herd** |
| --- | --- | --- |
| Dairy cows | Adult female cattle kept primarily for milking |  |
| Suckler cows | Adult female cattle kept primarily for rearing calves |  |
| Replacement heifers | Young female cattle (>10 months old and before first calving) intended for breeding |  |
| Calves  1-10 months old | All calves (male or female) 1-10 months old |  |
| Calves  Under 1 month old | All calves (male or female) <1 month old |  |
| Fatteners and stores | Cattle (male or female) intended for meat production (>10 months old) |  |
| Bulls | Adult male cattle kept for breeding |  |
| Other cattle | Any other cattle kept on the farm that do not fit any of the above categories. |  |

Please circle the correct responses **for each year** for every question shown in the table below.

|  |  | **2007** | | **2008** | | **2009** | | **2010** | |
| --- | --- | --- | --- | --- | --- | --- | --- | --- | --- |
| 2.2 | Were any cattle (of any type/age) purchased into the herd? | Yes | No | Yes | No | Yes | No | Yes | No |
| 2.3 | Were any shared bulls (bulls co-owned by this farm and at least one other farm) used in the herd? | Yes | No | Yes | No | Yes | No | Yes | No |
| 2.4 | Were any hired/borrowed cattle (including hired/borrowed bulls) brought into the herd? | Yes | No | Yes | No | Yes | No | Yes | No |
| 2.5 | Did any cattle visit and return from a show? | Yes | No | Yes | No | Yes | No | Yes | No |
| 2.6 | Did any cattle leave the herd and return for any other reason (e.g. market etc.)? | Yes | No | Yes | No | Yes | No | Yes | No |

**Questions 3 and 4**

| 3.1 | Were sheep kept on this farm at any time during the years shown **(circle correct responses)**: | **2007** | Yes | No |
| --- | --- | --- | --- | --- |
|  |  | **2008** | Yes | No |
|  |  | **2009** | Yes | No |
|  |  | **2010** | Yes | No |

Please **circle the correct responses** in the table below to indicate whether there were any contacts between cattle kept on this farm and the other specified livestock (Yes, No, Not Known (NK)). If there were contacts, **circle** the types of contacts (Direct (D) or Indirect (I)). More than one type of contact can be circled. If there were no contacts, circle No. If you are unsure if there were any contacts, circle NK.

Examples of Direct and Indirect contacts:

Direct contacts: Any event which may have led to physical contact between animals.

Examples: - Shared grazing.

- Nose-to-nose contact over fences.

- Straying of animals/breach of farm boundary.

Indirect contacts: Any means by which cattle in this herd could have come into contact with other animals via an intermediate source.

Examples: - Shared facilities and equipment.

- Shared personnel.

- Manure, slurry use.

|  |  | **2007** | | | **2008** | | | **2009** | | | **2010** | | |
| --- | --- | --- | --- | --- | --- | --- | --- | --- | --- | --- | --- | --- | --- |
| 3.2 | Contact with sheep on **this** farm | **Yes** | D | I | **Yes** | D | I | **Yes** | D | I | **Yes** | D | I |
|  |  | **No** | N/A | N/A | **No** | N/A | N/A | **No** | N/A | N/A | **No** | N/A | N/A |
|  |  | **NK** | N/A | N/A | **NK** | N/A | N/A | **NK** | N/A | N/A | **NK** | N/A | N/A |
| 4.1 | Contact with sheep on **other** farms | **Yes** | D | I | **Yes** | D | I | **Yes** | D | I | **Yes** | D | I |
|  |  | **No** | N/A | N/A | **No** | N/A | N/A | **No** | N/A | N/A | **No** | N/A | N/A |
|  |  | **NK** | N/A | N/A | **NK** | N/A | N/A | **NK** | N/A | N/A | **NK** | N/A | N/A |
| 4.2 | Contact with cattle on **other** farms | **Yes** | D | I | **Yes** | D | I | **Yes** | D | I | **Yes** | D | I |
|  |  | **No** | N/A | N/A | **No** | N/A | N/A | **No** | N/A | N/A | **No** | N/A | N/A |
|  |  | **NK** | N/A | N/A | **NK** | N/A | N/A | **NK** | N/A | N/A | **NK** | N/A | N/A |

**Question 5 – Calf information**

| 5.1 | What is the official ear tag no. of the calf?  (enter ‘0’ in first cell if calf not ear-tagged) |  |  |  |  |  |  |  |  |  |  |  |  |  |  |
| --- | --- | --- | --- | --- | --- | --- | --- | --- | --- | --- | --- | --- | --- | --- | --- |

| 5.2 | What is the calf’s date of birth? | D | D | M | M | Y | Y | Y | Y |
| --- | --- | --- | --- | --- | --- | --- | --- | --- | --- |

| 5.3 | Calf sex: **(circle correct response)** | Bull | Castrated male | Heifer |
| --- | --- | --- | --- | --- |
|  |  |

| 5.4 | Did the calf receive colostrum in the first  week of life? **(circle correct response)** | Yes | No | Unknown |
| --- | --- | --- | --- | --- |
|  |

**If yes**;

| What was the source of colostrum? **(circle all that apply)** | Own dam | Pooled natural colostrum from this herd | Commercial colostrum substitute | Other |
| --- | --- | --- | --- | --- |

**If other** circled, please describe:

**If commercial colostrum**

**substitute** circled, please

provide brand:

| 5.5 | Calf’s housing since birth:  **(circle all that apply)** | Calf hutch | Housed  (excluding calf hutch) | Outdoors |
| --- | --- | --- | --- | --- |
|  |  |  | | |

**Question 6 – Dam and sire information**

| 6.1 | What is the purpose of the calf’s dam? **(circle correct response)** | Dairy cow | Suckler cow |
| --- | --- | --- | --- |
|  |  | (see question 2.1 for description of dairy and suckler cows) | |

| 6.2 | What is the official ear tag no.  of the calf’s dam? |  |  |  |  |  |  |  |  |  |  |  |  |  |  |
| --- | --- | --- | --- | --- | --- | --- | --- | --- | --- | --- | --- | --- | --- | --- | --- |

| 6.3 | What breed is the calf’s dam? |  |
| --- | --- | --- |

| 6.4 | What breed is the calf’s sire? |  |
| --- | --- | --- |

| 6.5 | What is the date of birth of the calf’s dam? | D | D | M | M | Y | Y | Y | Y |
| --- | --- | --- | --- | --- | --- | --- | --- | --- | --- |

| 6.6 | Was the calf’s dam born on this farm? **(circle correct response)** | Yes | No |
| --- | --- | --- | --- |

**If No**;

| When did the calf’s dam arrive on this farm? | D | D | M | M | Y | Y | Y | Y |
| --- | --- | --- | --- | --- | --- | --- | --- | --- |

Please **circle the correct responses** in the table below to indicate whether there was any contact between this calf’s dam and the other specified livestock during the dam’s pregnancy with this calf. If there were contacts, **circle** the types of contacts (Direct (D) or Indirect (I)). More than one type of contact can be circled. If there were no contacts, circle No. If you are not sure if there were contacts, circle Not known.

|  | |  | | **Types of contact** | |
| --- | --- | --- | --- | --- | --- |
| 6.7 | Contact with sheep kept on **this** farm during the dam’s pregnancy? | | Yes | D | I |
|  |  |  | No |  |  |
|  |  |  | Not known |  |  |
| 6.8 | Contact with sheep kept on **other** farms during the dam’s pregnancy? | | Yes | D | I |
|  |  |  | No |  |  |
|  |  |  | Not known |  |  |
| 6.9 | Contact with cattle kept on **other** farms during the dam’s pregnancy? | | Yes | D | I |
|  |  |  | No |  |  |
|  |  |  | Not known |  |  |

**Question 7 – Vaccination history of the dam**

| 7.1 | While present in this herd has the calf’s dam ever been vaccinated against any disease? **(circle correct response)** | Yes | No |
| --- | --- | --- | --- |

**If answer to 7.1 is yes**;

Indicate by **circling the correct responses** whether, whilst present in this herd, the calf’s dam was vaccinated against the diseases shown in the table below in each of the years listed. If the dam was not present in this herd during a particular year, strike through the year in the first column and the rest of the row (do not strike through the year and rest of the row if the dam was present in the herd for part of the year). **It is imperative that complete and accurate data on vaccination are obtained. To facilitate collection of accurate data regarding administration of vaccines to the dam, the farm’s medicines record book MUST be scrutinised by the VIO during the interview process.** **If a Yes response in the table below is based on evidence other than the farm’s medicines record book (e.g. interviewee recall, invoices etc), and has not been corroborated by a corresponding record in the farm’s medicine book, please draw a diagonal line through the circled Yes response.**

| **Year** | **Calf’s dam vaccinated against:** | | | | | | | | | | | | | | |
| --- | --- | --- | --- | --- | --- | --- | --- | --- | --- | --- | --- | --- | --- | --- | --- |
|  | **Bluetongue** | | | **BVD** | | | **IBR** | | | **Leptospirosis** | | | **Other Disease** | | |
| **2005** |  |  |  | **Yes** | **No** | **Not known** | **Yes** | **No** | **Not known** | **Yes** | **No** | **Not known** | **Yes** | **No** | **Not known** |
| **2006** |  |  |  | **Yes** | **No** | **Not known** | **Yes** | **No** | **Not known** | **Yes** | **No** | **Not known** | **Yes** | **No** | **Not known** |
| **2007** |  |  |  | **Yes** | **No** | **Not known** | **Yes** | **No** | **Not known** | **Yes** | **No** | **Not known** | **Yes** | **No** | **Not known** |
| **2008** | **Yes** | **No** | **Not known** | **Yes** | **No** | **Not known** | **Yes** | **No** | **Not known** | **Yes** | **No** | **Not known** | **Yes** | **No** | **Not known** |
| **2009** | **Yes** | **No** | **Not known** | **Yes** | **No** | **Not known** | **Yes** | **No** | **Not known** | **Yes** | **No** | **Not known** | **Yes** | **No** | **Not known** |
| **2010** | **Yes** | **No** | **Not known** | **Yes** | **No** | **Not known** | **Yes** | **No** | **Not known** | **Yes** | **No** | **Not known** | **Yes** | **No** | **Not known** |

**If answer to 7.1 is yes;**

Use the table on the next seven pages to record information on vaccine brands administered to the calf’s dam whilst present in this herd (from 1 January 2005 onwards). If the dam was not present in this herd during a particular year, strike through the year in the column heading on the first page of the table (do not strike through the year if the dam was present in the herd for part of the year). **It is imperative that complete and accurate data on vaccines used including batch numbers and dates administered are obtained. To facilitate collection of accurate data on administration of vaccines to the dam, as noted above, the farm’s medicines record book MUST be scrutinised by the VIO during the interview process**. **Do not record a vaccine in the following table if information on the specific brand used is not available. If use of a specific vaccine brand on a particular date is based on evidence other than the farm’s medicines record book (e.g. interviewee recall, invoices etc), and has not been corroborated by a corresponding record in the farm’s medicine book, please indicate this by circling the date entry in the table**. **If a date entered in the following table is an estimate, please indicate this by writing E after the date entry (e.g. if the medicine book says ‘March 2009’ the date should be recorded as 01/03/2009 E). All vaccines used in the dam in the stated years should be recorded in the table (including any not already listed in the table).**

(√ ) – tick this column if the specified vaccine was administered to the dam during the stated year.

Date(s) – provide the date(s) the vaccine was administered to the calf’s dam during the stated year (specific day(s) if possible).

BN - provide the batch number(s) of the vaccine(s) used if available.

If the dam was not present in this herd during a particular year, strike through the year in the column heading on this page (do not strike through the year if the dam was present in the herd for part of the year).

| **Vaccine** | **Year** | | | | | | | | | | | |
| --- | --- | --- | --- | --- | --- | --- | --- | --- | --- | --- | --- | --- |
|  | **2005** | | **2006** | | **2007** | | **2008** | | **2009** | | **2010** | |
|  | **√** | **Date(s)** | **√** | **Date(s)** | **√** | **Date(s)** | **√** | **Date(s)** | **√** | **Date(s)** | **√** | **Date(s)** |
| Bluetongue vaccines |  | | | | | | | | | | | |
| **Bovilis BTV8** (Intervet Schering Plough) |  |  |  |  |  |  |  | DD: MM: 08  BN:  DD: MM: 08  BN:  DD: MM: 08  BN: |  | DD: MM: 09  BN:  DD: MM: 09  BN:  DD: MM: 09  BN: |  | DD: MM: 10  BN:  DD: MM: 10  BN:  DD: MM: 10  BN: |
| **BTV PUR Alsap 8** (Merial) |  |  |  |  |  |  |  | DD: MM: 08  BN:  DD: MM: 08  BN:  DD: MM: 08  BN: |  | DD: MM: 09  BN:  DD: MM: 09  BN:  DD: MM: 09  BN: |  | DD: MM: 10  BN:  DD: MM: 10  BN:  DD: MM: 10  BN: |
| **Zulvac 8 Bovis** (Fort Dodge/Pfizer) |  |  |  |  |  |  |  | DD: MM: 08  BN:  DD: MM: 08  BN:  DD: MM: 08  BN: |  | DD: MM: 09  BN:  DD: MM: 09  BN:  DD: MM: 09  BN: |  | DD: MM: 10  BN:  DD: MM: 10  BN:  DD: MM: 10  BN: |

| **Vaccine** | **Year** | | | | | | | | | | | |
| --- | --- | --- | --- | --- | --- | --- | --- | --- | --- | --- | --- | --- |
|  | **2005** | | **2006** | | **2007** | | **2008** | | **2009** | | **2010** | |
|  | √ | **Date(s)** | **√** | **Date(s)** | **√** | **Date(s)** | **√** | **Date(s)** | **√** | **Date(s)** | **√** | **Date(s)** |
| BVD vaccines |  | | | | | | | | | | | |
| **Bovidec** (Novartis) |  | DD:MM:05  BN:  DD:MM:05  BN:  DD:MM 05  BN: |  | DD: MM: 06  BN:  DD: MM: 06  BN:  DD: MM: 06  BN: |  | DD: MM: 07  BN:  DD: MM: 07  BN:  DD: MM: 07  BN: |  | DD: MM: 08  BN:  DD: MM: 08  BN:  DD: MM: 08  BN: |  | DD: MM: 09  BN:  DD: MM: 09  BN:  DD: MM: 09  BN: |  | DD: MM: 10  BN:  DD: MM: 10  BN:  DD: MM: 10  BN: |
| **Bovilis BVD** (Intervet Schering Plough) |  | DD:MM:05  BN:  DD:MM:05  BN:  DD:MM 05  BN: |  | DD: MM: 06  BN:  DD: MM: 06  BN:  DD: MM: 06  BN: |  | DD: MM: 07  BN:  DD: MM: 07  BN:  DD: MM: 07  BN: |  | DD: MM: 08  BN:  DD: MM: 08  BN:  DD: MM: 08  BN: |  | DD: MM: 09  BN:  DD: MM: 09  BN:  DD: MM: 09  BN: |  | DD: MM: 10  BN:  DD: MM: 10  BN:  DD: MM: 10  BN: |
| **Pregsure BVD** (Pfizer) |  | DD:MM:05  BN:  DD:MM:05  BN:  DD:MM 05  BN: |  | DD: MM: 06  BN:  DD: MM: 06  BN:  DD: MM: 06  BN: |  | DD: MM: 07  BN:  DD: MM: 07  BN:  DD: MM: 07  BN: |  | DD: MM: 08  BN:  DD: MM: 08  BN:  DD: MM: 08  BN: |  | DD: MM: 09  BN:  DD: MM: 09  BN:  DD: MM: 09  BN: |  | DD: MM: 10  BN:  DD: MM: 10  BN:  DD: MM: 10  BN: |

| **Vaccine** | **Year** | | | | | | | | | | | | |
| --- | --- | --- | --- | --- | --- | --- | --- | --- | --- | --- | --- | --- | --- |
|  | **2005** | | **2006** | | **2007** | | **2008** | | **2009** | | | **2010** | |
|  | √ | **Date(s)** | **√** | **Date(s)** | **√** | **Date(s)** | **√** | **Date(s)** | **√** | **Date(s)** | | **√** | **Date(s)** |
| IBR vaccines |  | | | | | | | | | | | | |
| **Bovilis IBR**  (Intervet Schering Plough) |  | DD:MM:05  BN:  DD:MM:05  BN:  DD:MM 05  BN: |  | DD: MM: 06  BN:  DD: MM: 06  BN:  DD: MM: 06  BN: |  | DD: MM: 07  BN:  DD: MM: 07  BN:  DD: MM: 07  BN: |  | DD: MM: 08  BN:  DD: MM: 08  BN:  DD: MM: 08  BN: |  | | DD: MM: 09  BN:  DD: MM: 09  BN:  DD: MM: 09  BN: |  | DD: MM: 10  BN:  DD: MM: 10  BN:  DD: MM: 10  BN: |
| **Bovilis IBR Marker Live** (Intervet Schering Plough) |  | DD:MM:05  BN:  DD:MM:05  BN:  DD:MM 05  BN: |  | DD: MM: 06  BN:  DD: MM: 06  BN:  DD: MM: 06  BN: |  | DD: MM: 07  BN:  DD: MM: 07  BN:  DD: MM: 07  BN: |  | DD: MM: 08  BN:  DD: MM: 08  BN:  DD: MM: 08  BN: |  | | DD: MM: 09  BN:  DD: MM: 09  BN:  DD: MM: 09  BN: |  | DD: MM: 10  BN:  DD: MM: 10  BN:  DD: MM: 10  BN: |
| **Rispoval IBR – Marker Inactivated** (Pfizer) |  | DD:MM:05  BN:  DD:MM:05  BN:  DD:MM 05  BN: |  | DD: MM: 06  BN:  DD: MM: 06  BN:  DD: MM: 06  BN: |  | DD: MM: 07  BN:  DD: MM: 07  BN:  DD: MM: 07  BN: |  | DD: MM: 08  BN:  DD: MM: 08  BN:  DD: MM: 08  BN: |  | | DD: MM: 09  BN:  DD: MM: 09  BN:  DD: MM: 09  BN: |  | DD: MM: 10  BN:  DD: MM: 10  BN:  DD: MM: 10  BN: |
| **Rispoval IBR – Marker Live** (Pfizer) |  | DD:MM:05  BN:  DD:MM:05  BN:  DD:MM 05  BN: |  | DD: MM: 06  BN:  DD: MM: 06  BN:  DD: MM: 06  BN: |  | DD: MM: 07  BN:  DD: MM: 07  BN:  DD: MM: 07  BN: |  | DD: MM: 08  BN:  DD: MM: 08  BN:  DD: MM: 08  BN: |  | | DD: MM: 09  BN:  DD: MM: 09  BN:  DD: MM: 09  BN: |  | DD: MM: 10  BN:  DD: MM: 10  BN:  DD: MM: 10  BN: |

| **Vaccine** | **Year** | | | | | | | | | | | |
| --- | --- | --- | --- | --- | --- | --- | --- | --- | --- | --- | --- | --- |
|  | **2005** | | **2006** | | **2007** | | **2008** | | **2009** | | **2010** | |
|  | √ | **Date(s)** | **√** | **Date(s)** | **√** | **Date(s)** | **√** | **Date(s)** | **√** | **Date(s)** | **√** | **Date(s)** |
| **Leptospirosis vaccines** |  | | | | | | | | | | | |
| **Leptavoid H** (Intervet Schering Plough) |  | DD:MM:05  BN:  DD:MM:05  BN:  DD:MM 05  BN: |  | DD: MM: 06  BN:  DD: MM: 06  BN:  DD: MM: 06  BN: |  | DD: MM: 07  BN:  DD: MM: 07  BN:  DD: MM: 07  BN: |  | DD: MM: 08  BN:  DD: MM: 08  BN:  DD: MM: 08  BN: |  | DD: MM: 09  BN:  DD: MM: 09  BN:  DD: MM: 09  BN: |  | DD: MM: 10  BN:  DD: MM: 10  BN:  DD: MM: 10  BN: |
| **Spirovac** (Pfizer) |  | DD:MM:05  BN:  DD:MM:05  BN:  DD:MM 05  BN: |  | DD: MM: 06  BN:  DD: MM: 06  BN:  DD: MM: 06  BN: |  | DD: MM: 07  BN:  DD: MM: 07  BN:  DD: MM: 07  BN: |  | DD: MM: 08  BN:  DD: MM: 08  BN:  DD: MM: 08  BN: |  | DD: MM: 09  BN:  DD: MM: 09  BN:  DD: MM: 09  BN: |  | DD: MM: 10  BN:  DD: MM: 10  BN:  DD: MM: 10  BN: |

| **Vaccine** | **Year** | | | | | | | | | | | |
| --- | --- | --- | --- | --- | --- | --- | --- | --- | --- | --- | --- | --- |
|  | **2005** | | **2006** | | **2007** | | **2008** | | **2009** | | **2010** | |
|  | √ | **Date(s)** | **√** | **Date(s)** | **√** | **Date(s)** | **√** | **Date(s)** | **√** | **Date(s)** | **√** | **Date(s)** |
| Other Vaccines | If a vaccine has been used in the dam that is not already listed below, please add to the list stating the specific vaccine name (full brand details) and manufacturer. Please only list vaccines if information on the specific brand used is available. | | | | | | | | | | | |
| **Bovivac S** (Intervet Schering Plough) |  | DD:MM:05  BN:  DD:MM:05  BN:  DD:MM 05  BN: |  | DD: MM: 06  BN:  DD: MM: 06  BN:  DD: MM: 06  BN: |  | DD: MM: 07  BN:  DD: MM: 07  BN:  DD: MM: 07  BN: |  | DD: MM: 08  BN:  DD: MM: 08  BN:  DD: MM: 08  BN: |  | DD: MM: 09  BN:  DD: MM: 09  BN:  DD: MM: 09  BN: |  | DD: MM: 10  BN:  DD: MM: 10  BN:  DD: MM: 10  BN: |
| **Enviracor** (Pfizer) |  | DD:MM:05  BN:  DD:MM:05  BN:  DD:MM 05  BN: |  | DD: MM: 06  BN:  DD: MM: 06  BN:  DD: MM: 06  BN: |  | DD: MM: 07  BN:  DD: MM: 07  BN:  DD: MM: 07  BN: |  | DD: MM: 08  BN:  DD: MM: 08  BN:  DD: MM: 08  BN: |  | DD: MM: 09  BN:  DD: MM: 09  BN:  DD: MM: 09  BN: |  | DD: MM: 10  BN:  DD: MM: 10  BN:  DD: MM: 10  BN: |
| **Lactovac** (Pfizer) |  | DD:MM:05  BN:  DD:MM:05  BN:  DD:MM 05  BN: |  | DD: MM: 06  BN:  DD: MM: 06  BN:  DD: MM: 06  BN: |  | DD: MM: 07  BN:  DD: MM: 07  BN:  DD: MM: 07  BN: |  | DD: MM: 08  BN:  DD: MM: 08  BN:  DD: MM: 08  BN: |  | DD: MM: 09  BN:  DD: MM: 09  BN:  DD: MM: 09  BN: |  | DD: MM: 10  BN:  DD: MM: 10  BN:  DD: MM: 10  BN: |

| **Vaccine** | **Year** | | | | | | | | | | | |
| --- | --- | --- | --- | --- | --- | --- | --- | --- | --- | --- | --- | --- |
|  | **2005** | | **2006** | | **2007** | | **2008** | | **2009** | | **2010** | |
|  | √ | **Date(s)** | **√** | **Date(s)** | **√** | **Date(s)** | **√** | **Date(s)** | **√** | **Date(s)** | **√** | **Date(s)** |
| **Rotavec Corona** (Intervet Schering Plough) |  | DD:MM:05  BN:  DD:MM:05  BN:  DD:MM 05  BN: |  | DD: MM: 06  BN:  DD: MM: 06  BN:  DD: MM: 06  BN: |  | DD: MM: 07  BN:  DD: MM: 07  BN:  DD: MM: 07  BN: |  | DD: MM: 08  BN:  DD: MM: 08  BN:  DD: MM: 08  BN: |  | DD: MM: 09  BN:  DD: MM: 09  BN:  DD: MM: 09  BN: |  | DD: MM: 10  BN:  DD: MM: 10  BN:  DD: MM: 10  BN: |
| **Trivacton 6** (Merial) |  | DD:MM:05  BN:  DD:MM:05  BN:  DD:MM 05  BN: |  | DD: MM: 06  BN:  DD: MM: 06  BN:  DD: MM: 06  BN: |  | DD: MM: 07  BN:  DD: MM: 07  BN:  DD: MM: 07  BN: |  | DD: MM: 08  BN:  DD: MM: 08  BN:  DD: MM: 08  BN: |  | DD: MM: 09  BN:  DD: MM: 09  BN:  DD: MM: 09  BN: |  | DD: MM: 10  BN:  DD: MM: 10  BN:  DD: MM: 10  BN: |
|  |  | DD:MM:05  BN:  DD:MM:05  BN:  DD:MM 05  BN: |  | DD: MM: 06  BN:  DD: MM: 06  BN:  DD: MM: 06  BN: |  | DD: MM: 07  BN:  DD: MM: 07  BN:  DD: MM: 07  BN: |  | DD: MM: 08  BN:  DD: MM: 08  BN:  DD: MM: 08  BN: |  | DD: MM: 09  BN:  DD: MM: 09  BN:  DD: MM: 09  BN: |  | DD: MM: 10  BN:  DD: MM: 10  BN:  DD: MM: 10  BN: |
|  |  | DD:MM:05  BN:  DD:MM:05  BN:  DD:MM 05  BN: |  | DD: MM: 06  BN:  DD: MM: 06  BN:  DD: MM: 06  BN: |  | DD: MM: 07  BN:  DD: MM: 07  BN:  DD: MM: 07  BN: |  | DD: MM: 08  BN:  DD: MM: 08  BN:  DD: MM: 08  BN: |  | DD: MM: 09  BN:  DD: MM: 09  BN:  DD: MM: 09  BN: |  | DD: MM: 10  BN:  DD: MM: 10  BN:  DD: MM: 10  BN: |

| **Vaccine** | **Year** | | | | | | | | | | | |
| --- | --- | --- | --- | --- | --- | --- | --- | --- | --- | --- | --- | --- |
|  | **2005** | | **2006** | | **2007** | | **2008** | | **2009** | | **2010** | |
|  | √ | **Date(s)** | **√** | **Date(s)** | **√** | **Date(s)** | **√** | **Date(s)** | **√** | **Date(s)** | **√** | **Date(s)** |
|  |  | DD:MM:05  BN:  DD:MM:05  BN:  DD:MM 05  BN: |  | DD: MM: 06  BN:  DD: MM: 06  BN:  DD: MM: 06  BN: |  | DD: MM: 07  BN:  DD: MM: 07  BN:  DD: MM: 07  BN: |  | DD: MM: 08  BN:  DD: MM: 08  BN:  DD: MM: 08  BN: |  | DD: MM: 09  BN:  DD: MM: 09  BN:  DD: MM: 09  BN: |  | DD: MM: 10  BN:  DD: MM: 10  BN:  DD: MM: 10  BN: |
|  |  | DD:MM:05  BN:  DD:MM:05  BN:  DD:MM 05  BN: |  | DD: MM: 06  BN:  DD: MM: 06  BN:  DD: MM: 06  BN: |  | DD: MM: 07  BN:  DD: MM: 07  BN:  DD: MM: 07  BN: |  | DD: MM: 08  BN:  DD: MM: 08  BN:  DD: MM: 08  BN: |  | DD: MM: 09  BN:  DD: MM: 09  BN:  DD: MM: 09  BN: |  | DD: MM: 10  BN:  DD: MM: 10  BN:  DD: MM: 10  BN: |
|  |  | DD:MM:05  BN:  DD:MM:05  BN:  DD:MM 05  BN: |  | DD: MM: 06  BN:  9DD: MM: 06  BN:  DD: MM: 06  BN: |  | DD: MM: 07  BN:  DD: MM: 07  BN:  DD: MM: 07  BN: |  | DD: MM: 08  BN:  DD: MM: 08  BN:  DD: MM: 08  BN: |  | DD: MM: 09  BN:  DD: MM: 09  BN:  DD: MM: 09  BN: |  | DD: MM: 10  BN:  DD: MM: 10  BN:  DD: MM: 10  BN: |
|  |  | DD:MM:05  BN:  DD:MM:05  BN:  DD:MM 05  BN: |  | DD: MM: 06  BN:  DD: MM: 06  BN:  DD: MM: 06  BN: |  | DD: MM: 07  BN:  DD: MM: 07  BN:  DD: MM: 07  BN: |  | DD: MM: 08  BN:  DD: MM: 08  BN:  DD: MM: 08  BN: |  | DD: MM: 09  BN:  DD: MM: 09  BN:  DD: MM: 09  BN: |  | DD: MM: 10  BN:  DD: MM: 10  BN:  DD: MM: 10  BN: |

**Question 8 – Dam’s diet**

| 8.1 | Which of the following feed types were provided to the calf’s dam during the 12 months prior to calving this calf and the one month after calving this calf? **(circle correct responses in the table below)** | | | |
| --- | --- | --- | --- | --- |
|  | | **Feed Provided?** | |  |
| **Grass** | | Yes | No |  |
| **Grass silage (all types)** | | Yes | No |  |
| **Maize silage** | | Yes | No |  |
| **Straw** | | Yes | No |  |
| **Hay** | | Yes | No |  |
| **Manufactured concentrate, protein supplement or meal** | | Yes | No |  |
| **Salt or mineral** | | Yes | No |  |
| **Other** | | Yes | No |  |

If ‘other’ feed types were provided please list in table below:

| **Other feed(s) – Home produced** | **Other feed(s) – Purchased** |
| --- | --- |
|  |  |
|  |  |
|  |  |
|  |  |
|  |  |
|  |  |
|  |  |

If a manufactured concentrate, protein supplement or meal was provided please list in the table below:

| **Product name** | **Manufacturer** |
| --- | --- |
|  |  |
|  |  |
|  |  |
|  |  |
|  |  |
|  |  |
|  |  |

# DATA TRANSFER PERMISSION FORM

By signing this form, the cattle herd owner (or the owner’s representative) is authorising the Veterinary Laboratories Agency to have access to veterinary medicines sales records held for this herd by the herd’s private veterinary surgeon for the years 2003 to 2010 inclusive.

CATTLE HERD DETAILS

Trading name:

Owner’s name:

Address:

Signature: Print name:

Date:

PRIVATE VETERINARY SURGEON DETAILS

Trading name:

Contact name:

Address:

Telephone no.

VETERINARY LABORATORIES AGENCY DETAILS

Trading name: Veterinary Laboratories Agency

Contact name: Mr A Holliman

Contact address: VLA Penrith, Merrythought, Calthwaite, Penrith, Cumbria CA11 9RR

Telephone no: 01768 885295

### Data Protection Act 1998

Defra is the data controller of any personal information supplied to the Veterinary Laboratories Agency (VLA). VLA, an Executive Agency of Defra, may use this information and test results for anonymous disease surveillance. This includes looking for new or emerging diseases in the UK and monitoring changes in disease patterns.

While strictly maintaining client confidentiality, Defra (including the VLA) and others will use the data to produce reports and publications. In the interest of public health and animal health, it may be necessary to share data (including contact details) with the Food Standards Agency, Health Protection Agency, Veterinary Medicines Directorate, other Defra Executive Agencies, local authorities and other agencies/organisations. They may use this information to contact you in relation to protecting public and animal health.

For animals in the UK at the time of sampling, VLA will report positive results for notifiable diseases, along with all relevant data, to Animal Health, an Executive Agency of Defra and, where appropriate, to devolved administrations. This is in line with our legal obligations under the Animal Health Act 1981. Animal Health will use this information in their efforts to control the disease.

Defra/VLA may need to release information, including personal and commercial information, on request, under the Environmental Information Regulations, or the Freedom of Information Act 2000. However, Defra/VLA will not allow any unwarranted breach of confidentiality nor will we contravene our obligations under the Data Protection Act 1998. VLA may use the name, address and other details provided to contact you about occasional customer research aimed at improving our services.

(Amended: July 2009)

For more information about how data is handled see the Data Handling Guarantee.

**Subsequent to completion and signing by the herd owner (or owner’s representative), please provide the herd owner with a photocopy of pages 16 and 17 of this questionnaire.**
